# Healthcare Data for Achieving a More Personalized Treatment of Chronic Kidney Disease

**DOI:** 10.3390/biomedicines9050488

**Published:** 2021-04-29

**Authors:** Francisco Herrera-Gómez, F. Javier Álvarez

**Affiliations:** 1 Kidney Resuscitation and Acute Purification Therapies, Complejo Asistencial de Zamora, Sanidad de Castilla y León, 49022 Zamora, Spain; 2 Transplantation Center, Lausanne University Hospital & University of Lausanne, CH-1011 Lausanne, Switzerland; 3 Pharmacological Big Data Laboratory, Faculty of Medicine, University of Valladolid, 47005 Valladolid, Spain; alvarez@med.uva.es

**Keywords:** real-world evidence, chronic kidney disease, biomarkers

## Abstract

The current concept of healthcare incites a more personalized treatment of diseases. To this aim, biomarkers are needed to improve decision-making facing chronic kidney disease (CKD) patients. Prognostic markers provided by real-world (observational) evidence are proposed in this Special Issue entitled “Biomarkers in Chronic Kidney Disease”, with the intention to identify high-risk patients. These markers do not target measurable parameters in patients but clinical endpoints that may be in turn transformed to benefits under the effect of future interventions.

Healthcare is changing. Currently and more and more, there is an urgent need for targeted or more personalized therapies, which should be especially addressed to the most susceptible patient populations. Shifting the focus from a one-size-fits-all system to one that is patient-tailored, must provide clinicians with the right tools to treat the right patient with the right dose of the right medicine (or medicines) at the right time. In this context, biomarkers should allow the evaluation and improve the impact of the interventions destined to chronic kidney disease (CKD) patients, with the objective to achieve the most clinically rewarding outcomes for them [1]. Importantly, CKD patients are a diverse (heterogenous) population that exhibit the features of a susceptible patient population. Indeed, CKD is the end-of-way for a constellation of diseases and serious conditions such as diabetes, hypertension, metabolic syndrome, high cardiovascular risk, etc., which impose particular support for such individuals.

A complicated decision-making process may be facilitated if specific markers for CKD patients are available. The common markers used in non-CKD populations are not useful for managing individuals with kidney function deterioration. A paradoxical effect from such markers leads to confusion, it is already noticeable among patients with moderated kidney function deterioration, and clinically clear in the end-stage of kidney disease (ESKD) [2].

According to the definition of the National Institute for Health (NIH), a biomarker is “a characteristic that is objectively measured and evaluated as an indicator of normal biological processes, pathogenic processes or pharmacological responses to a therapeutic intervention” [3]. This broad definition allows the consideration as biomarkers a large variety of parameters (from levels of small molecules/presence of cellules up to physical measurements including imaging data). It does not, however, characterize prognostic markers such as those proposed by the studies published in our Special Issue.

In addition, biomarkers that are easily available in everyday practice and understandable to clinicians who adjust treatments should especially be preferred. In fact, in many of the most important clinical areas, user-friendly biomarkers are not available, probably as a consequence of the current momentum and enthusiasm for more complex markers.

## 1. Real-World Evidence Biomarkers for Chronic Kidney Disease

Facing the current situation and to boost translational epidemiology (1), this Special Issue presents a collection of observational studies providing real-world evidence of accessible-for-all risk factors with an interest to treat CKD patients specifically. Approximately 10 years ago, an interesting review contextualized the importance of observational evidence to reach a personalized treatment period for CKD [4]. As an initiative, well-performed research, developed by the teams of Professors Soulillou and Brouard from Nantes, France, pioneers and contributes still to improve outcomes of kidney recipients [5,6].

Real-world (observational) data constitutes a source of information to evaluate (evidently less rigorously if compared to clinical trials) diseases and patients affected by diseases. Large databases are now being performed to enlarge the knowledge on diseases and patients, and to facilitate physicians’ decisions. For instance, new risk factors for patients with coronavirus disease 2019 (COVID-19) and acute kidney function deterioration are proposed by our team, and such factors may be used as prognostic markers for this population to prevent bad outcomes [7]. In addition, there is an enormous potential into real-world evidence for its assessment in more complex analyses by observational evidence with clinical trial evidence in order to answer crucial questions, for example, on the topic of orphan drugs aiming to improve kidney outcomes [8].

## 2. Pragmatic Contributions

Since several years, the amino acid metabolite profile was found to be related to CKD apparition. Indeed, the kidneys directly manage circulating levels of such molecules. However, not only kidney function decline may be predicted by amino acid levels and their ratios, but the characteristics of kidney affectation may be reflected by given profiles [9]. For instance, an inflammatory background of CKD may be depicted by high indoleamine 2,3-dioxygenase (IDO) activity, which may be detected by an increased kynurenine/tryptophan ratio. Similarly, worse citrulline and kynurenine levels and a high kynurenine/tryptophan ratio may be expected in association with increasing proteinuria, which is associated commonly with bad outcomes in CKD. Therefore, efforts in the daily management of patients should be addressed to identify and treat high-risk CKD patients, and not only be limited to the prediction of the reduction in the estimated glomerular filtration rate (eGFR).

The intensity of inflammation and its consequences may in turn be measured. Oxidative stress, uremia, malnutrition and a high risk for cardiovascular events are expressions of the same common “magnum” alteration, which contribute to high CKD-associated mortality, as well as to morbidity charge as eGFR progressively deteriorates. Recently, there is an interest to better know the association between advanced glycation end-product (AGE) levels and soluble forms of the receptor for AGE (RAGE) with the pro-inflammatory state of patients with CKD. Particularly, this Special Issue highlights the inverse association between the cleave RAGE/endogenous secretory RAGE ratio with the risk of death in CKD patients [10]. Therefore, high-risk (“inflamed”) CKD patients may now be easily identified in the clinic to improve their treatment in order to prevent (or slow down the occurrence of) bad outcomes such as cardiovascular events, need of dialysis, etc. We are confident that other markers of the pro-inflammatory state of CKD patients (related or not with AGE products) may and will be proposed, and the literature on this is increasing. For instance, loss of muscle mass demonstrated by sarcopenia is in relation to the increase in AGE products, which is interesting in diabetic CKD by their links with insulin resistance and other alterations in diabetes mellitus [11]. A vicious circle, and a way without return, now must be in the future a treatable condition preventing the progression of CKD. Regardless of nutritional recommendations, pharmacological interventions such as antioxidants, modulators of chronic inflammation are desired.

Interestingly, biomarkers must be looked for anywhere in CKD patients, i.e., all potential sources should be considered, and observations should not only be centered in metabolites. For instance, changes in gut microbiome composition towards a state of “intestinal dysbiosis”, contributing to a pro-inflammatory environment, should be searched in early CKD, especially in the cases of diabetic CKD or when a metabolic disease is suspected as the cause of CKD [12]. Future research should propose therapeutic options addressed to prevent an acquired dysbiotic microbiome, as current options exist to limit the impact of many other risk factors influencing the progression of CKD (e.g., antihypertensive medications, statins for treating dyslipemia, anti-proteinuria interventions, etc.).

## 3. Strategies for Managing Data and Their Limits

Analyses leading to proposed biomarkers may be forced to consider composite outcomes (e.g., more clinically significant outcomes such as the progression status of CKD considering the start of dialysis or kidney transplantation are more profitable instead to consider only kidney function deterioration), especially when using groups of markers are presumed to be more efficacious than using separately given biomarkers (even in the case of molecules with a good predictive value) [13]. Especially, non-linear kidney function decline forces more complex assessments in order to propose well-performing tools to predict the progression of CKD, as well as other outcomes among CKD patients that may all necessitate calculations following elaborated dichotomous contexts formulae [14]. In this way, comorbidities leading to CKD are now considered by models in which the calculated effect size determines the real impact of such comorbidities. In any case, the research collected by this Special Issue is not focused on the measurable parameters of patients but on the clinical endpoints that may be in turn transformed to benefits under the effect of future interventions.

Nevertheless, observational evidence and especially that provided by real-world environments are limited importantly by confounders, among other biases such as attrition, selection and that related to information. It should not be forgotten that researchers on the “real-world” (mostly clinicians and other actors of the healthcare milieu) are working outside rigorous research environments considering randomization to guarantee a more credible research production. In this context, sample size may oblige to repeat assessments in order to reproduce observations, and to make that then generalizable to the main population affected by CKD.

## 4. Concluding Remarks

Observational evidence is becoming an interesting way to propose biomarkers for persons affected by CKD, considering its diversity (heterogeneity) and the several conditions conducing to and maintaining kidney function loss and alterations associated to that. Examples of well-performing research are increasing in recent years, even if limitations in this type of evidence are criticizable when compared to more formal research dominated by randomization. The examples presented in this Special Issue aim to identify high-risk CKD and propose objectives targetable by future interventions.
